# Transcranial approach to pituitary adenomas invading the cavernous sinus: A modification of the classical technique to be used in a low-technology environment

**DOI:** 10.4103/2152-7806.65054

**Published:** 2010-07-01

**Authors:** Aldo Spallone, Roberto V. Vidal, Justo G. Gonzales

**Affiliations:** 1 Department of Clinical Neurosciences, Section of Neurosurgery, Neurological Centre of Latium “NCL”, Rome, Italy; 2 Calixto Garcia University Hospital, La Havana, Cuba; 3 Division of Neurosurgery, Hermanos Amejeiras Hospital, La Havana, Cuba

**Keywords:** Cavernous sinus surgery, fronto-orbito-zygomatic craniotomy (FOZ), Hakuba’s triangle, invasive adenoma, transcranial approach

## Abstract

**Objective:**

Pituitary adenomas invading the cavernous sinus represent a therapeutic challenge. Those tumors have been traditionally treated with incomplete surgical removal, observation and/ or adjunctive medical therapy, and radiotherapy. In relatively recent years, some authors have suggested a main direct surgical approach to cavernous sinus (CS) with the aim of complete removal of the adenoma, either by a modified trans-sphenoidal route, using or not an endoscopy-assisted approach, or by a transcranial direct approach. The latter has the advantage of allowing direct exposure of the lesion with a view of the surgical field unhindered by important neurovascular structures.

**Materials and Methods:**

We report a technical modification of the classical epidural approach for CS adenoma removal. This was used in 14 patients. Surgical technique included a fronto-orbito-zygomatic craniotomy with extradural anterior clinoidectomy, and intradural approach to the Hakuba’s triangle for intracavernous dissection. The tumors were removed under direct vision.

**Results:**

Total macroscopical removal was achieved in all but one case. This patient required postoperative radiation therapy as well as adjuvant dopaminergic regime for achieving control of preoperatively increased hormonal values. No other case required radiotherapy. Hormonal and/ or clinical control was also achieved in all the remaining cases. Out of the remaining 13 cases, all appeared to be tumor free at an average postoperative observation at 78 months (34 to 90 months). Significant surgical sequels were detected in only 1 case (persistent 3^rd^ nerve palsy and moderate hemiparesis).

**Conclusions:**

This experience, though limited, would suggest that the transcranial limited CS exposure through the Hakuba’s triangle may allow adequate removal of intracavernous pituitary adenomas with very good long-term results and acceptable complication rate.

## INTRODUCTION

Surgical management of pituitary adenomas is, as a rule, performed using the trans - sphenoidal route. However, 3% to 7% of pituitary adenomas grow laterally out of the sellar boundaries,[10,26,32,34] and a significant proportion of these tumors invade the cavernous sinus (CS) and represent a management dilemma and difficult-to-treat lesions.

In fact, invasion of the CS has been a reason for considering these lesions not amenable to radical surgery, at least until recent years,[11] and for including postoperative radiation and/ or hormonal therapy in the gold standard of the management of patients harboring these, though histologically benign, tumors.[16,23,42]

In relatively recent years, dissection of CS has been introduced mostly by Dolenc[11,12] in the technical armamentarium of modern neurosurgery, and attempts by several authors[2,13,24,33,40,41] to radically remove pituitary adenomas invading the CS have been successful basically the technique described originally by Dolenc. Other authors[8,14,19,31] have reported their experiences with widened inferior approaches, more recently with the aid of endoscopy,[15] again with the aim of maximizing tumor removal. Nevertheless, the majority of pituitary surgeons still prefer to accomplish incomplete trans-sphenoidal removal of an invasive adenoma and to use in some cases postoperative radiotherapy (or better still, radiosurgery[21,29]) for obtaining control of residual tumor.

This reluctance to attempt radical surgical removal of these lesions is based on the consideration that adenoma, as a rule, does not present with an aggressive biological behavior; however, it is perhaps also related to the fact that direct CS surgery is a very demanding task. Actually inferior CS approaches would oblige the surgeon to work in very limited, deep, narrow spaces, where the view is hindered by crucial neurovascular structures. Endoscopy can help in overcoming this latter problem[4,15] but cannot solve the former, i.e., the narrowness of the anatomical spaces and the consequent limited view.[5,10,25] Moreover, in an environment where modern neurosurgical technology could be somehow lacking due to economical restraints, neuroendoscopy would not be a possible priority. On the other hand, transcranial dissection, either sub-epidural or epidural,[11,12,18] is technically a demanding, not risk-free surgical maneuver, which can be considered routine only in very experienced hands.[12,22]

In an 8-year period (1997-2005), we managed 17 cases of pituitary adenoma with definite invasion of CS. The first 3 cases were operated using the epidural approach described by Dolenc[11,12] and Kawase.[18] All these cases showed the expected complete oculomotor paralysis following surgery, which however regressed 3 to 6 months after operation.

The remaining 14 cases were operated using a modification of the subdural approach, which made, in our opinion, the surgery significantly easier though apparently not less effective than the original technique superbly illustrated and recently revised by Dolenc.[12] This technique is described here.

## MATERIALS AND METHODS

In the 6-year period covered by the present study (1999-2005), 14 patients harboring a pituitary adenoma invading the CS were operated upon either in the Nuova Clinica Latina (presently Neurological Centre of Latium, NCL), Rome, Italy; or in the H. Amejeiras Hospital, La Havana, Cuba.

There were 10 women and 4 men aged 24 to 49 years (average, 40.5 years). The presenting symptoms were mostly headache, amenorrhea-galactorrhea and decreased libido. Total preoperative ophthalmoplegia was observed in 2 patients [Table 1]. The tumors’ endocrinological characteristics are summarized in Table 2. Preoperative and postoperative hormonal values were checked with radioimmunoassay, and reference values are also specified in the table.

**Table 1:** Preoperative and postoperative symptoms

Preoperative symptom	Cured		Improved		*n*° variation		Total	
	
	*n*°	%	*n*°	%	*n*°	%	*n*°	%
Headache	6	66,6	3	33,4	0	0	9	100
Amenorrhea	3	42,9	3	42,9	1	14,2	7	100
Galactorrhea	5	83,3	1	16,7	0	0	6	100
Decreased libido	3	50,0	2	33,3	1	16,7	6	100
Visual defect	0	0	3	100	0	0	3	100
Ophthalmoplegia	2	100	0	0	0	0	2	100
Epilepsy	1	100	0	0	0	0	1	100
Acral growth	0	0	1	100	0	100	1	100

**Table 2:** Preoperative and postoperative endocrinological status

	*n*° Patients	Preoperative	Postoperative
PRL-secreting	5	min 72- max 96 (Av. 88)	min 13- max 25 (Av.19)
Cortisol-secreting	3	min 42 – max 66 Av. 51)	min 6 – max 6 (Av: 11)
GH-secreting	1	24	3
Cortisol-PRL-secreting	1	PRL 70 Cortisol 49	PRL 12 Cortisol 2
No secreting	4		
Total	12		

Reference values: PRL 2-15 ng/mL (electrochemiluminescent assay, ECLIA); Cortisol 5- 25 mcg/dL (immunoassay, MEIA); GH < 5 ng/mL (chemiluminescent assay)

**Figure 1:**
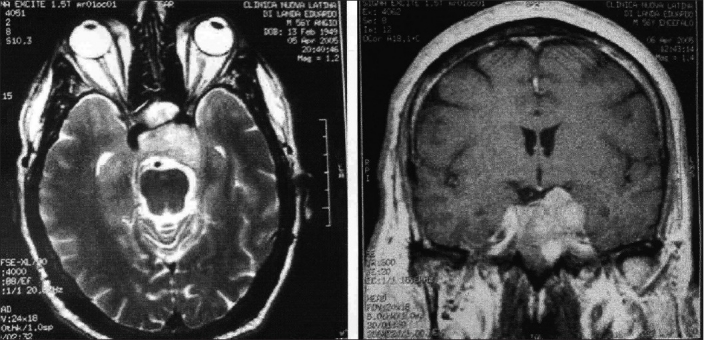
a) MRI scan, axial T2 image. A pituitary tumor invading the left CS and encasing the ICA is demonstrated. b) The T1 post-contrast coronal scan gives clear evidence of the extra-sellar lateral and superior extensions of the lesion

CT scan was performed in all the present cases; and MRI, in the most recent 10 cases. CS invasion was staged 3 in 8 cases, and 4 in 6 cases, according to the scale proposed by Knosp *et al*.,[20] in order to evaluate objectively the invasion of the CS [Figure 1]. In the 4 cases in which only CT scanning was performed, the internal carotid artery (ICA) represented the landmark for staging CS tumor invasion. This artery had close relationships with the lesion in all the present cases and was surrounded by the tumor in several of them.

The goal of surgical management was to achieve total removal of the lesion in order to allow discharging the patient without any adjuvant postoperative treatment whenever possible. Postoperative clinical and neuroimaging data were available for all patients with a follow-up of 34 to 103 months (average, 83.1 + 3.4 months). In particular, serial clinical and neuroimaging follow-ups were performed at regular intervals in the two Italian patients, whilst a recent follow-up evaluation was conducted during the second half of the year 2009 in all Cuban patients.

### Surgical technique

**Figure 2:**
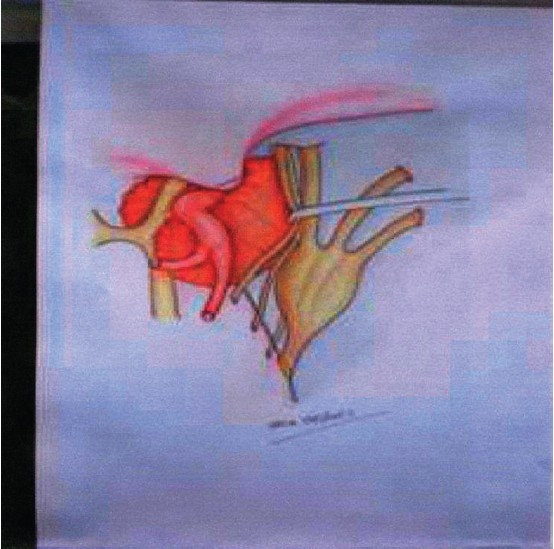
Drawing of the surgical anatomy. The intracavernous portion of the adenoma is fully exposed following lateral displacement of the released III cranial nerve using a nerve self-retaining retractor

A fronto-orbito-zygomatic (FOZ) approach, as described in a previous paper,[36] complemented by an extradural clinoidectomy, was used in all patients. The dura is opened in a ‘T’ fashion along the Sylvian fissure and the fronto-temporal base. Dural incision is prolonged in the direction of the dura propria of the optic nerve for approximately 2 cm (not to the orbit!). Ipsilateral optic nerve and the ICA are mobilized first, followed by contralateral optic nerve up to the chiasma. Tumor capsule is incised in the interoptic space, and the tumor is removed with suction, bipolar coagulation and microcurettes. Next, tumor is removed from the ipsilateral optico-carotid space; this maneuver is highly facilitated by the mobility of the neurovascular structures obtained, thanks to the approach. If the tumor has eroded the sellar opening, at this stage it is usually possible to enter the sella via the diaphragm from the interoptic space, and/ or along the ICA at the origin of the ophthalmic artery, using the corridors which the tumor has created during its expansion. Unavoidably, some blind maneuvers, careful ones, using microcurettes and curved suckers are obligatory. However, the final result of these maneuvers as far as radicality of tumor removal can be checked with a mirror (or an endoscope) inserted into the sella via a widened sellar opening from the interoptic space. If this is not possible due to limited sellar opening, an intradural trans-sphenoidal approach as described by Patterson *et al*.[28] is performed. With this the sellar tumor is removed under direct vision, while visually controlling also the position of the contralateral ICA. Attention is directed next to the space lateral to the intradural ICA, which is also free from tumor. At this step, the III cranial nerve is widely exposed, and the posterior communicating artery also. Both these structures may have close relationships with the tumor and must be exposed. Then the III cranial nerve (CN) is mobilized from the porus oculomotorius to the orbit using a careful dissection with an arachnoidal knife. Some bleeding resulting from inadvertent opening of the CS close to the orbit is easily controlled with little pieces of Surgicel. When totally released, the III CN is retracted laterally using a nerve microretractor. In this manner the IV CN is handled, also laterally, without having to be exposed, and this widens laterally the opening of Hakuba’s triangle.[17] Intracavernous tumor is fully exposed in this manner and can be removed under direct vision [Figure 2]. The tumor pseudocapsule protects the underlying intracavernous ICA from injury, whilst bleeding from the CS is usually minimal, also due to limited dissection. Intracavernous tumor extension was not required *per se* in our cases for opening of the antero-medial triangle and consequent release of the dural ring for adequate ICA mobilization. After adequate hemostasis, fat grafts of convenient size are placed at the level of the cranial base and/ or in the sella. The T-shaped dural opening is closed with running suture, and the craniotomy closed in the usual manner. A lumbar drainage is left in place for 5 days. Early postoperative CT scanning is obtained for checking adequateness of the tumor removal and absence of relevant surgical complications.

### Early postoperative results

**Figure 3:**
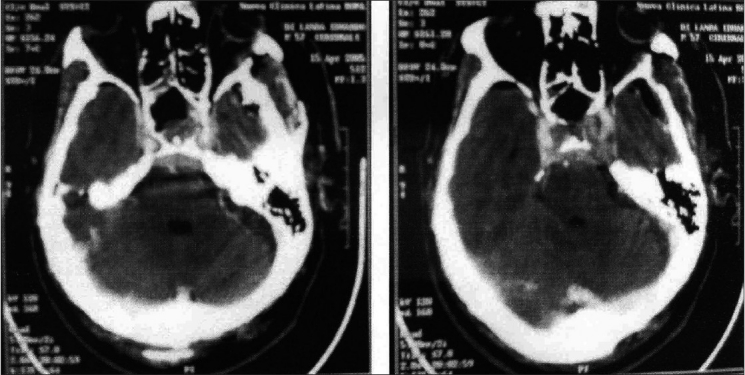
Same case as Figure 1. Immediate postoperative CT scan, before Figure 3a and after Figure 3b, contrast enhancement shows apparent total tumor removal

**Figure 4:**
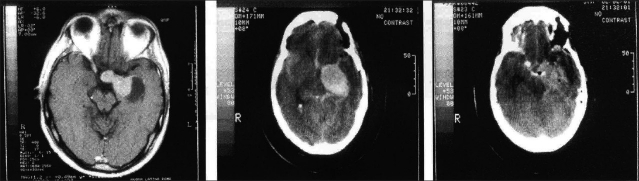
a) Axial T1 scan shows a pituitary tumor with significant left lateral extra-sellar extension. b) Immediate postoperative CT scan shows a huge hematoma. c) CT scan following re-operation demonstrates partial removal of the mass

There was no operative mortality. Total macroscopical tumor removal, as confirmed by immediate postoperative CT scanning [Figure 3], was achieved in all but one case. Immediate postoperative complications included newly occurring III CN palsy (6 cases), IV CN paresis (1 case), CSF leak (2 cases) and postoperative hematoma requiring surgical evacuation (1 case). Postoperative hematoma occurred in a patient who harbored a giant PRL-secreting highly fibrous adenoma expanding laterally under the MCA deep into the temporal lobe, which could be removed only up to 80% of its mass [Figures 4a–4c]. The one with the adenoma patient exhibited a definitive worsening of his preoperative hemiparesis, and total oculomotor palsy. Both complications regressed to some extent during the late postoperative course.

Postoperative persistent (lasting more than 2 days) diabetes insipidus was not noticed in any of the present patients.

### Late postoperative results

Immediate postoperative III CN palsy cleared within 2 months in all but one case. CSF leak required prolonged lumbar drainage and bed rest and resolved within 2 weeks in both patients in whom this complication had occurred [Table 3].

**Table 3:** Complications and sequels

Complication	*n*° Patients
III CN paresis	6
IV CN paresis	1
CSF leak	2
Hematoma	1
Sequels	*n*° Patients
III CN paresis	1
Hemiparesis	1

Routine follow-ups, either CT scanning (8 cases) and/ or MRI (6 cases), were done 6-84 months after operation in order to check for possible tumor recurrence.

**Figure 5:**
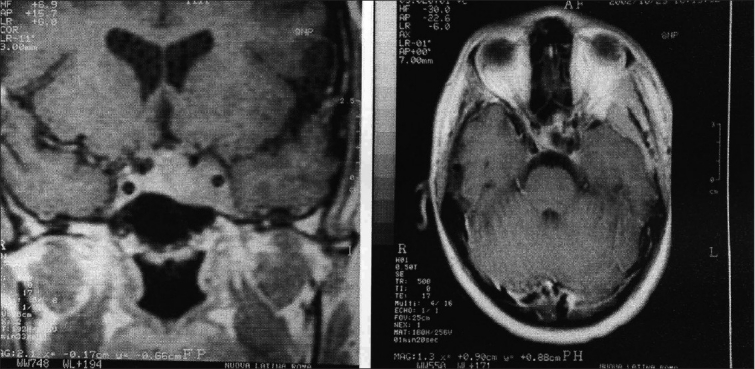
a) MRI, T1 post-contrast coronal scan shows a Knosp 3-4 intracavernous adenoma. b) Postoperative MRI, T1 axial scan. This demonstrates apparent total removal of the tumor

No case exhibited tumor remnants [Figures 5a and 5b], with the exception of the patient with partial tumor removal [Figure 4], who was given postoperative conventional radiation therapy. This lesion was checked with periodical CT controls and appeared to be stable under medical DA treatment 60 months following surgery, when the patient died due to an acute gastrointestinal illness, apparently unrelated to his pituitary adenoma and complicated by mesenteric venous thrombosis. After postsurgical recovery, the patient, though mildly handicapped (Karnofsky 80), had been working.

**Figure 6:**
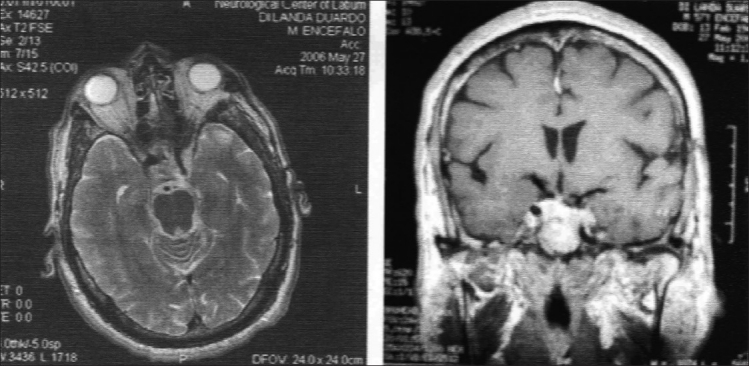
Same patient as in Figures 1 and 3. Two-year postoperative MRI. T2 axial. a) Post-contrast T1 coronal; b) Scans show no recurrence of the tumor

**Figure 7:**
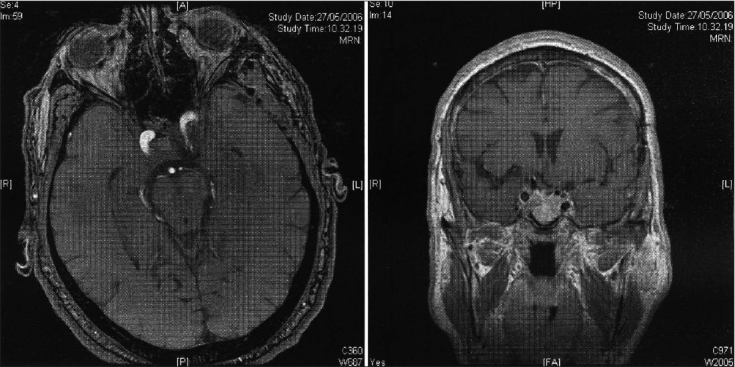
Same patient as in Figures 1, 3, 6. Five-year postoperative axial; a) coronal; Figure b) MRI shows no changes as compared to the earlier postoperative MRI controls

An Italian patient, harboring an ACTH-secreting adenoma and showing a total ophthalmoplegia preoperatively [Figures 1 and 3], showed excellent hormonal control following an apparently radical surgery, which matched well with the postoperative imaging [Figures 6a and 6b]. At the 4-year follow-up, in spite of the stabile neuroimaging examinations, which did not indicate a possible recurrence [Figures 7a and 7b], a recurrent though mild elevation of the ACTH (179 pg/mL; normal range, 5-60 pg/mL) was noticed, whilst cortisol was within the range. The patient, also following the suggestions of his endocrinologist, considered prophylactic, lately administrated radiation therapy. Interestingly, a further checkup conducted 4 months later showed completely normal results, and recommendation for radiotherapy was withdrawn. He is now well and still with his ACTH in the normal range, 12 months after that episode. Another patient, a 50-year-old man harboring at the same time a Parkinson’s disease, was mildly incapacitated due to the latter (Karnofsky 90); however, he showed no problems related to his PRL-secreting totally removed adenoma. One patient, harboring a non-secreting tumor, died from unrelated causes (myocardial infarction) 34 months following removal of a non-secreting tumor. He had been in good health since then.

Diabetes insipidus was not recorded as a long-term complication. Control of increased preoperative hormonal levels was obtained in all but 3 patients, all of them harboring a PRL-secreting adenoma; however, serum PRL values were only mildly increased and significantly less than the preoperative ones. It should be noted that only 1 of these patients, the one who died of mesenteric thrombosis, was placed under DA treatment following surgery. One patient who had an ACTH-secreting tumor exhibited signs of mild hypopituitarism (FSH level, 0.3 *µ*L/L). Four cases of non-secreting adenoma have shown no signs of recurrence until now. The remaining 6 cases showed no signs of increased hormonal values when last checked, 80 to 94 (average, 78) months after operation.

Table 4 summarizes the 5-year postoperative evolution of the Karnofsky scale of the present patients.

**Table 4:** Karnofsky scale follow-up

Karnofsky Scale	Patient follow-up							
	Discharge		6 months		1 year		5 years	
	*n*°	%	*n*°	%	*n*°	%	*n*°	%
70	1	7.2	1	7,2	0	0	0	0
80	2	14,2	1	7,2	3	21.5	1	7.7
90	4	28,6	5	35	3	21.5	4	30.8
100	7	50,0	7	50,0	8	57.0	8	61.5
Total	14	100	14	100	14	100	13	100
Mean	92,1		92,8		93.5		95.3	TOT

## DISCUSSION

### General considerations

Pituitary adenomas invading the CS are rare, at least those tumors with clinically and/ or radiologically relevant CS invasion. They are detected mostly in female adults.[1,7,20,39] A female predominance was noted also in the present cases, as there was also predominance of PRL-secreting adenomas.

Dolenc[12] has pointed out that the III cranial nerve possesses a surprising capacity of resisting a long-term though significant tumor compression. However, this does not prevent patients with adenomas invading the CS from showing clinical symptoms of oculomotor dysfunction, very significant in some cases.[12,41] Two of our 14 patients exhibited a total oculomotor palsy before surgery, which regressed in both cases within a few months after surgery.

Hormonal dysfunctions and headache represented the chief complaint in the present cases, whilst visual disturbances were under-represented when compared with other statistics.[12]

As suggested by recent literature,[7,9,20,23,27,30,39] MRI represented the main diagnostic test. We used the scale proposed by Knosp *et al*.[20] for evaluating objectively CS tumor invasion.

All patients in the present study represented a high-grade (3 or 4) case according to this classification.

### Management philosophy

As a rule, routine trans-sphenoidal surgery does not allow radical removal of a CS pituitary adenoma.[10,12,25] Incompletely removed adenomas do not necessarily grow further, and close postoperative observation can be a reasonable clinical strategy.

Medical therapy with DA, and possibly also somatostatin, may be effective in controlling hormone-secreting pituitary adenomas.

Radiotherapy can be also administrated for incompletely removed pituitary adenomas.[16,34] Although this treatment is not free of complications, also relevant ones such as radiation-induced tumors,[3,12,35] there is no doubt that it can be effective. Modern radiosurgery is certainly capable of giving at least the same results as those of traditional radiation therapy, with fewer complications. However, complications can occur also with radiosurgery for CS adenomas,[21,29] and this must be carefully balanced against the risk of radical surgery.

The present experience was a result of our working mainly in an environment where modern LINAC radiotherapy was available in only a few centers, where quite logically priority was given to patients harboring malignant tumors. We decided that, it was worthwhile trying to cure these lesions with radical surgery, these being CS adenoma benign lesions, and we acted accordingly. This philosophy is obviously hardly recommendable on a routine basis in a high-tech environment.

Widened inferior approaches did not appear to have significantly increased the possibility of radical removal of a pituitary tumor invading the CS,[14] although a more recently published statistic seems to indicate that a fair number of intracavernous adenomas can be eradicated using an extended trans-sphenoidal approach.[19] Endoscopic trans-sphenoidal surgery was recently advocated for increasing surgical radicality in the removal of CS adenomas.[15] However, the main problem related to such an approach, i.e., narrowness and deepness of the surgical field, has been also recently pointed out in an elegant anatomical study performed by a distinguished group of pioneers in endoscopic pituitary surgery.[5] It is quite possible that advancement in endoscopic technology and increasing surgical experience with direct endoscopic CS adenomas surgery would render other, apparently more invasive techniques obsolete. However, this is not yet the present scenario, as honestly recognized also by the aforementioned group of internationally reputed experts on endoscopic pituitary surgery.[5]

Our experience with craniobasal approaches[36,37] has led us to consider mainly a transcranial approach for removing pituitary adenomas invading the CS. It should he noted that craniotomy can be performed without sophisticated drilling technology[37]; whilst also anterior clinoidectomy, which we perform using a high-speed drill, can be performed with routine bone-working instruments, as recently suggested by Chang.[6]

### Surgical technique

The technique described in this report was the result of the natural evolution of our surgical experience during the course of 6 years. Our group’s philosophy included objective evaluation of postoperative results. As far as CS pituitary adenomas are concerned, early experience led us to change our approach from an epidural one to the one here described due to the following considerations:

a- Epidural CS dissection gave nice exposure of the bulging intracavernous adenoma, as a rule, between the III and the IV cranial nerves; however, the sellar content could not be visualized (maybe also due to lack of experience). This led to early tumor recurrence in 2 cases (as we have stated above) operated upon with an epidural approach and not included in the present study.

b- Epidural dissection invariably led to significant bleeding, particularly when dissecting at the level of the confluence of the Sylvian veins.[18] This forced us to pack significantly the CS.

c- Extended CS dissection requires release of the dural ring (a not risk-free maneuver unless performed by extremely experienced surgeons) and mostly, as a rule, more CS packing.

The facts mentioned above under b- and c- together may well explain the routine occurrence of — though as a rule, temporary — oculomotor palsy in the cases operated upon using an epidural CS dissection, which to our surprise was detected in only 50% of the CS adenoma patients operated upon using the technique herein described. The occurrence of III nerve palsy is obviously a major problem for the patient. However, this is, as a rule, temporary, lasts approximately only 3 months and is commonly present preoperatively.

Basically, we attempted as much as possible to simplify the operative steps of CS dissection as described by master surgeons.[12,13,15,33] We are well aware that this can be criticized. In particular, we know that removal of the tuberculum sellar for obtaining a radical removal of the intrasellar portion of the adenoma can carry several risks, including meningitis as a consequence of CSF leak, or damage of the pituitary stalk. However, the latter never occurred in our experience; and CSF leak, a well-known risk in any Skull-base procedure, occurred in only 2 cases and was adequately managed with well-known remedies, viz., slight elevation of the head and prolonged lumbar drainage. We matched this risk against (1) the (admittedly low) risk of arterial injury during the surgical release of the dural ring, which we do not do in adenomas invading the CS, but also (2) the necessity of packing a bleeding CS in the case of extended dissection of the sinus. If only the Hakuba’s triange is opened bleeding is usually minor and packing is as a rule, unnecessary. Possibly also related to this, postoperative total oculomotor palsy occurred inconstantly. Moreover, adequate exposure of the sellar content protected from the risk of incidental injury of the contralateral ICA,[12] because all the relevant neurovascular structures were under direct view in the microsurgical field.

The present technique does not require extensive opening of the dura propria of the optic nerve, and this simplifies the dural closure. As stated above, using the precautions routinely utilized in craniobasal surgery — such as fat grafting complemented by fibrin glue, postoperative lumbar drainage and slight head elevation — was strict routine in our cases.

We were obliged to perform some blind maneuvers in order to remove portions of the tumor from hidden corners; however, this is routine in pituitary adenoma surgery. We want to stress that, as stated above, most relevant neurovascular structures were well exposed and consequently were under control when performing any blind maneuver for tumor removal.

We want to stress also that this technique does not require a sophisticated technological environment, but only a reasonably good microscope and a few micro-instruments, as well as good training in neurosurgical craniobasal anatomy. All these are concrete possibilities also in low-technology neurosurgical environments, as found routinely in less developed countries.

## RESULTS

Our study follow-up averages 82 months. Such a period of time does not allow definitive conclusions regarding possible tumor recurrence at a later stage, although such an evidence would seem to be extremely unlikely.[38]

Total macroscopical tumor removal, achieved in all but one case, was assessed by routine early postoperative CT scanning and late postoperative either CT scan and/ or MRI. This matched well with the positive evolution of clinical symptoms following the operation. In 1 case, increase in postoperative hormonal serum levels led us to consider prophylactic, lately administrated radiation therapy, in spite of the lack of signs of recurrence at the serial neuroimaging controls. Recommendation for radiotherapy was ultimately withdrawn following a further hormonal checkup 4 months later, at which ACTH and cortisol serum levels appeared to be well within the normal range.

Surgical cure of an invasive adenoma is always a challenge because these tumors spread extradurally without a real capsule, and neoplastic cells can be easily left behind.[22,26,32] However, at least until now, we have no evidence to conclude that surgery was not curative in the present cases, and consequently no reason for indicating routine postoperative radiation therapy. Postoperative evaluation of Karnofsky’s made in our patient was positive and compared favorably with the preoperative one.

Surgical complications were not negligible: 13% of patients had postoperative CSF leak; 50% had oculomotor palsy; in addition to a postoperative hematoma and related complications occurring, however, in an extremely difficult case. However, in all but one case, complications either regressed spontaneously, as in the case of oculomotor palsy, or were easily dealt with routine postoperative maneuvers, as in the case of CSF leak.

## CONCLUSIONS

Pituitary adenomas invading the CS represent a challenge for neurosurgeons. However, several treatment modalities are presently available, including preoperative medical treatment with the aim of reducing the tumor in size in order to make it amenable to radical trans-sphenoidal surgery; and radiosurgery, whether or not preceded by partial surgical removal.

However, it must not be forgotten that these are benign tumors, possibly amenable to surgical cure. On the basis of this consideration, some authors have described their experiences with direct approaches to CS, either from below or transcranially, and have recommended these approaches. All the techniques used for possible radical surgery have obvious advantages and disadvantages, as expected, but also have in common the peculiarity of being definitely technically demanding ones. We have described here a transcranial approach that does not necessarily require sophisticated equipment, can be used also in a relatively less sophisticated technological environment and apparently make cavernous sinus adenoma surgery a little less demanding when compared with the classically described techniques for CS surgical dissection; this approach, at the same time, allows the adenoma to be, at least apparently, totally removed in the vast majority of cases. This is particularly a relevant issue in a low-tech environment and/ or in a developing country, where postoperative adjuvant therapy cannot be easily administered to any patient, and regular follow-ups are relatively difficult. Certainly, endoscopic trans-sphenoidal removal of CS adenomas represents the future. However, the present result should be considered as a bottom line to be reached, as far as radicality of tumor removal by the new techniques of minimally invasive craniobasal surgery is concerned.
